# Syndrome sérotoninergique secondaire à l’association de paroxétine et amitriptyline: à propos d’un cas

**DOI:** 10.11604/pamj.2024.48.109.44113

**Published:** 2024-07-17

**Authors:** Kaouthar El Mir, Wiame EL Bouchalli, Salah-Eddine El Jabiry, Barrimi Mohammed

**Affiliations:** 1 Faculté de Médecine et de Pharmacie Oujda, Université Mohammed Premier, Oujda, Maroc,; 2 Service de Psychiatre, Centre Hospitalo-Universitaire Mohammed VI, Oujda, Maroc,; 3 Laboratoire de Recherche sur la Santé Materno-Infantile et Mentale, Faculté de Médecine et de Pharmacie Oujda, Université Mohammed Premier, Oujda, Maroc,; 4 Laboratoire d'Immuno-Hématologie et Thérapie Cellulaire, Faculté de Médecine et de Pharmacie Oujda, Université Mohammed Premier, Oujda, Maroc

**Keywords:** Syndrome sérotoninergique, inhibiteurs sélectifs de recapture de sérotonine, paroxétine, amitriptyline, cas clinique, Serotonin syndrome, selective serotonin reuptake inhibitors, paroxetine, amitriptyline, case report

## Abstract

Le syndrome sérotoninergique est dû essentiellement à une cause iatrogénique par un médicament sérotoninergique, caractérisé par un polymorphisme symptomatique qui peut le faire confondre avec d´autres situations cliniques retardant le diagnostic et mettant en jeu le pronostic vital, à la lumière d´un cas clinique et d´une revue de la littérature, nous rapportons le cas clinique d´un patient qui a présenté un état de confusion avec une agitation psychomotrice et une hyperréfléxie et des myoclonies spontanées suite à la prise simultanée de la paroxétine et l´amitriptyline. Le diagnostic de syndrome sérotoninergique a été retenu après avoir éliminé les autres éventuels diagnostics, nécessitant une hospitalisation en unité de soins intensifs. Cette présentation clinique souligne l'importance de bien connaître les manifestations cliniques du syndrome sérotoninergique pour assurer une prise en charge thérapeutique précoce et adaptée.

## Abstract (en)

Serotonin syndrome is an iatrogenic disorder induced by serotonergic drug. It is characterised by a symptomatic polymorphism that may create confusion with other clinical conditions, delaying diagnosis and putting the vital prognosis at risk. In light of a case report and a literature review, we here report the clinical case of a patient presenting with confusion, psychomotor agitation, hyperreflexia and spontaneous myoclonus following simultaneous use of paroxetine and amitriptyline. The diagnosis of serotonin syndrome was made after ruling out other possible diagnoses. The patient was admitted to the intensive care unit. This clinical case highlights the importance of thoroughly understanding the clinical manifestations of serotonin syndrome to ensure early and appropriate therapeutic management.

## Introduction

Le syndrome sérotoninergique se caractérise par un ensemble de manifestations cliniques potentiellement fatales, secondaires à un excès de sérotonine (5-hydroxytryptamine [5-HT]) au niveau du système nerveux central et périphérique. La majorité des cas de syndrome sérotoninergique survient dans un cadre iatrogénique, souvent suite à une association de médicaments à effet sérotoninergique, les cas de syndrome sérotoninergique en monothérapie étant plus rares [1]. Il peut toucher toute tranche d´âge et son incidence est en augmentation du fait de l´élévation de la prévalence de la dépression et l´utilisation des antidépresseurs notamment les inhibiteurs sélectifs de la recapture de la sérotonine [2]. Ce syndrome est probablement sous-estimé par les cliniciens à cause de son tableau clinique initial polymorphe et non spécifique, ce qui rend le diagnostic difficile à établir [3].

Nous rapportons et nous discutons le cas clinique d´un patient qui présentait un syndrome sérotoninergique suite à la prise concomitante de la paroxétine et l´amitriptyline afin d´éclairer l´intérêt une bonne connaissance de la manifestation clinique du syndrome sérotoninergique.

## Patient et observation

**Informations du patient:** il s´agit de Mr RL, âgé de 27 ans, soudeur de profession, célibataire, de bas niveau socioéconomique et sans antécédent pathologique particulier.

**Résultats cliniques:** le patient était confus (*Glasgow Coma Scale (GCS)* 13/15-ème), agité pupilles en mydriase, avait des tremblements avec tachypnée et tachycardie, une hyperréfléxie associée à des myoclonies spontanées plus marquées au niveau des membres inférieurs avec une hypersudation et des frissons.

**Chronologie:** l´histoire de maladie remonte à une semaine avant sa consultation chez un médecin neurologue en secteur libéral, par l´installation des propos de persécution, des propos de négation de filiation, une bizarrerie de comportement et des attitudes hallucinatoires puis le tableau s´est compliqué par une agitation psychomotrice et un diagnostic initial d´un accès psychotique aigu a été retenu, et le patient a été mis sous halopéridone 5 mg par jour.

Deux mois après, l´évolution a été marquée par une mauvaise observance thérapeutique avec persistance de la symptomatologie psychotique associée à des symptômes négatifs tel que la perte de plaisir, l´isolement et l´abolition avec un sommeil perturbé, motivant le malade à refaire une consultation chez le même médecin traitant qui lui a ajouté de la paroxétine 20 mg par jour et l´amitriptyline 25 mg par jour.

Quelques heures (6 à 8h) après la prise des deux antidépresseurs, le malade présentait une perte de conscience et agitation psychomotrice motivant son hospitalisation aux unités des soins intensifs.

**Démarche diagnostique:** le malade a bénéficié d´un bilan biologique complet, objectivant une perturbation des électrolytes avec une acidose métabolique et un taux de créatine phosphokinase (CPK) à 100 UL/L, l'examen cytobactériologique des urines (ECBU), l'étude du liquide céphalorachidien et les hémocultures se sont avérés négatifs. La concentration plasmatique de la *C-reactive protein (CRP)* et la numération formule sanguine étaient normales.

L´électrocardiogramme (ECG) a objectivé une arythmie cardiaque par fibrillation auriculaire, et la radiographie thoracique et l'imagerie par résonance magnétique (IRM) cérébrale étaient sans particularités. Le diagnostic de syndrome sérotoninergique a été retenu après avoir éliminé une étiologie infectieuse, métabolique ou neurologique.

**Intervention thérapeutique:** le patient a été mis sous ventilation mécanique, gavage 500cc/8H, réhydratation par eau plate 500cc/8H, ration de base SG 5% + 2g Nacl + 2g Kcl +1g ca 2+ / 8h , et SS 0,9% 500cc/4H, sédation par midazolam 8mg/H, IPP 40mg /j, avec un anticoagulant à visée préventive et insuline selon dextro.

**Suivi et résultats:** l´état clinique hémodynamique et neurologique du patient s´est stabilisé après 24h, ensuite il a été transféré au service de psychiatrie où le médecin traitant a trouvé un patient calme sur le plan moteur, bien orienté dans le temps et l´espace, ayant eu un délire mystico religieux et de persécution, certaines réponses déplacées, un jugement perturbé sans présence d´un syndrome dépressif ni maniaque ou confusionnel. L´évolution de la symptomatologie psychiatrique a été marquée par une bonne amélioration avec un bon retour à l´état pré morbide, stabilisée sous neuroleptique atypique type olanzapine avec une posologie initiale de 5 mg par jour.

**Point de vue du patient:** le patient a été très satisfait de notre prise en charge et souhaiterait partager son expérience avec d'autres patients.

**Consentement éclairé:** le malade nous a confié son consentement éclairé pour que son cas soit publié tout en gardant son anonymat.

## Discussion

Le syndrome sérotoninergique survient en raison d'une augmentation de la transmission et de l'activité sérotoninergiques par une stimulation excessive des récepteurs sérotoninergiques. Il présente une importante variabilité inter-individuelle. Le polymorphisme des cytochromes P450 pourrait être une des explications à cette variabilité. Dans le cadre du syndrome sérotoninergique, seule l´inhibition enzymatique est potentiellement responsable du syndrome sérotoninergique. Prenant notre cas, il y avait une association de risque élevé de syndrome sérotoninergique qui constitue de l´imipramine (amitriptyline) qui représente le substrat des cychromes P450 2D6 avec la paroxétine qui est un inhibiteur puissant à ce sous type de cytochrome et cela peut expliquer la survenue du syndrome sérotoninergique [4].

Les manifestations cliniques du syndrome sérotoninergique se caractérisent par un triade symptomatique: une altération de l´état mental: agitation, anxiété, désorientation, excitation; anomalie neuromusculaire: tremblements, clonies, hyperréfléxie, rigidité musculaire, akathisie; l'hyperactivité du système neurovégétatif: hypertension, tachycardie, tachypnée, mydriase, muqueuses sèches, frisson, ou hyperthermie, vomissement, diarrhée, bruits intestinaux hyperactifs, arythmies.

L´installation des symptômes est rapide et parfois brutale, et survient majoritairement dans les 6 heures à 24 heures suivant l´introduction ou le changement de médication [5], comme c´était le cas de notre patient dont il présentait initialement une confusion mentale, une agitation, un mydriase, une tachycardie et tachypnée, hyperréfléxie avec un clonus des membres inférieurs puis le tableau est compliqué par un trouble de conscience, trouble de rythme cardiaque, trouble hydroélectrolytique et insuffisance respiratoire.

Le diagnostic positif du syndrome sérotoninergique se base essentiellement sur les critères diagnostiques de Hunter qui sont des nouveaux critères, plus simples, plus sensibles (84% vs. 75%) et plus spécifiques (97% vs. 96%) que les critères de Dunkley *et al*. [6], ils reposent essentiellement sur: la prise d´un médicament sérotoninergique et la présence d´un de ces 5 symptômes suivants: tremblement, hyperréfléxie; clonus spontané (membres inférieurs+); rigidité musculaire, température au-delà de 38°C + clonus oculaire ou inductible (à la dorsiflexion+); clonus oculaire + agitation ou sueurs; clonus inductible + agitation ou sueurs.

De nombreux diagnostics différentiels peuvent se confondre avec le diagnostic du syndrome sérotoninergique, comprennent le syndrome malin des neuroleptiques, l'hyperthermie maligne, la toxicité des anticholinergiques. Notre patient ayant un des agents antipsychotiques en cause dans le syndrome malin des neuroleptiques, mais l´absence d´hyperthermie et de la rigidité musculaire, avec normalisation de taux de créatine kinase qui est souvent supérieur à 1000 UI/L dans le syndrome malin des neuroleptiques [7], tandis que le malade avait une installation brutale des symptômes, présence d´une association à haut risque (amitriptyline + paroxétine) avec une amélioration rapide après 24h de la mise en condition et un score de Naranjo est à 7 ce qui indique une probabilité élevée d'une relation causale entre l'administration des deux antidépresseurs et l'effet indésirable observé [8], ce qui faisait retenir le diagnostic du syndrome sérotoninergique. Nous rappelons dans le Tableau 1 les différents diagnostics différentiels du syndrome sérotoninergique [9].

**Tableau 1:** les diagnostics différentiels du syndrome sérotoninergique

Trouble	Agent incriminé	Début	Résolution	Signes cliniques
Syndrome sérotoninergique	Sérotonine	Débute dans 6h à 24h et parfois brutale	Disparait après 24h avec traitement	Confusion, rigidité musculaire, clonus spontané ou occulaire, agitation hyperréflexie, augmentation bruits intestinaux, hypersudation
Syndrome malin des neuroleptique	Antagoniste de dopamine	Débute dans quelques jours à quelques semaines	Après 9j avec traitement	Hypertension, tachycardie, tachypnée, hyperthermie; bradyreflexie; rigidité musculaire très sévere
Hyperthermie maligne	Inhalation des produits anesthésiques	Débute dans quelques minutes à 24h	24h à 48h avec traitement	Absence d’une hyperréflexie ou clonus; cyanose, rigidité; peau marbrée
Toxicité anticholinergique	Agents anticholinergiques	Débute dans les 24h	Quelques heures à quelques jours avec traitement	Reflexe et tonicité musculaire normale Rétention urinaire diminution des bruits intestinaux peau érythémateuse chaude et sèche

La prise en charge thérapeutique de syndrome sérotoninergique repose sur l´arrêt de toute médication se base sur un agoniste sérotoninergique, il peut souvent suffire à la résolution des symptômes, la surveillance des constantes vitales avec une mise en condition adéquate pour garder le pronostic vital, la gestion de l´agitation par les benzodiazépines, l´utilisation de cyproheptadine qui peut également servir de test diagnostique: uniquement par voie orale à la dose initiale de 12 mg dans les premières 24 heures, suivi de 4 à 8 mg toutes les 6 heures en cas de persistance des symptômes), ainsi que la chlorpromazine à la dose de 100 mg, si l´on est sûr du diagnostic. Concernant l´évolution la majorité des cas de syndrome sérotoninergique ayant habituellement une disparition des symptômes de 24 à 48 heures après l´arrêt des médicaments sérotoninergiques responsables, alors que le moindre des cas qui nécessitent une prise en charge lourde avec un risque de décès [10].

## Conclusion

Le syndrome sérotoninergique est une urgence diagnostique et thérapeutique, rare mais mortelle, de manifestations purement cliniques, ses caractéristiques sont polymorphes dont le médecin psychiatre doit être plus prudent pour ne pas le confondre avec les autres diagnostics différentiels d´autant plus le syndrome malin des neuroleptiques.
